# Body composition change during neoadjuvant chemotherapy for breast cancer

**DOI:** 10.3389/fonc.2022.941496

**Published:** 2022-08-26

**Authors:** Min Kyeong Jang, Seho Park, Chang Park, Ardith Z. Doorenbos, Jieon Go, Sue Kim

**Affiliations:** ^1^ Mo-Im Kim Nursing Research Institute, Yonsei University College of Nursing, Seoul, South Korea; ^2^ Division of Breast Surgery, Department of Surgery, Yonsei University College of Medicine, Seoul, South Korea; ^3^ Department of Biobehavioral Nursing Science, College of Nursing, University of Illinois Chicago, Chicago, IL, United States; ^4^ Department of Medicine, University of Illinois Cancer Center, Chicago, IL, United States

**Keywords:** body composition, breast neoplasm, muscle/skeletal, neoadjuvant chemotherapy, sarcopenia

## Abstract

**Background:**

Sarcopenia is receiving attention in oncology as a predictor of increased chemotherapy toxicities. Research into body composition change during neoadjuvant chemotherapy for breast cancer is both urgently needed and generally lacking. This study assessed sarcopenia prevalence before and after neoadjuvant chemotherapy using CT imaging, evaluated body composition changes during neoadjuvant chemotherapy, and determined predictors of sarcopenia status after neoadjuvant chemotherapy for breast cancer.

**Materials and Methods:**

In this retrospective, descriptive study, we used data collected from 2017 to 2020 to measure body composition parameters on cross-sectional CT slices for 317 Korean women with breast cancer patients before and at completion of neoadjuvant chemotherapy. Changes in skeletal muscle index, visceral fat index, subcutaneous fat index, and sarcopenia were assessed and correlated, and multivariate logistic regression was conducted to identify predictive factors associated with sarcopenia status at completion of neoadjuvant chemotherapy.

**Results:**

Of the 80 breast cancer patients (25.2%) who had sarcopenia before beginning neoadjuvant chemotherapy, 64 (80.0%) retained their sarcopenia status after chemotherapy. Weight, body mass index, body surface area, and visceral fat index showed significant increases after neoadjuvant chemotherapy; notably, only skeletal muscle index significantly decreased, showing a reduction of 0.44 cm^2^/m^2^ (*t* (316) = 2.15, *p* <.5). Lower skeletal muscle index at baseline was associated with greater loss of muscle mass during neoadjuvant chemotherapy (*r* = −.24, *p* <.001). Multivariate logistic regression showed that baseline sarcopenia status was the only significant predictor of sarcopenia status after neoadjuvant chemotherapy (*p* <.001). Specifically, the log odds of sarcopenia after neoadjuvant chemotherapy were 3.357 higher in the baseline sarcopenia group than in the group without baseline sarcopenia (β = 3.357, *p* <.001).

**Conclusion:**

Sarcopenia during neoadjuvant chemotherapy can be obscured by an increasing proportion of fat in body composition if clinical assessment focuses on only body mass index or body surface area rather than muscle mass. For breast cancer patients who have sarcopenia when they begin neoadjuvant chemotherapy, the risk of muscle mass loss during treatment is alarmingly high. To reduce masking of muscle mass loss during treatment, comprehensive evaluation of body composition, beyond body surface area assessment, is clearly needed.

## Introduction

Breast cancer, with an estimated 2.1 million cases diagnosed globally in 2018 (1), is one of the most prevalent cancers in women. A recent trend analysis showed an increasing trend in breast cancer incidence rates, especially among younger women, but a downward trend in mortality rates (2). With increasing survival rates, the disease burden of breast cancer survivors is also increasing worldwide. In the search for effective strategies to improve long-term management of physical and psychological consequences of cancer and its treatment in this population, previous studies have emphasized the need to improve body composition. For example, one study showed that wearable technology to improve body composition (3) produced a significant reduction in body fat, weight, and BMI. Furthermore, a previous study that applied a 4-week rehabilitation protocol for breast cancer survivors showed significant reduction of fatigue and improvement of muscle mass and function (4). In addition, a previous systematic review that focused on cancer treatment-induced bone loss in patients with early breast cancer identified medicinal treatments that improved bone mass density but also called for further bone health management (5). These studies indicate that better treatment outcomes are achievable for breast cancer survivors, but to accomplish this, assessments of risk factors for poor clinical outcomes arising during cancer treatment are important.

Sarcopenia, or decreased muscle mass, has been receiving attention in oncology as a predictor of increased chemotherapy toxicities and as a sensitive early marker of treatment effectiveness (6–9). The prevalence of sarcopenia among breast cancer patients has been variously reported as 45.0% and as ranging from 15.9% to 66.9% (10). In addition, sarcopenia has been related to a significantly higher risk of mortality. In a study of 1,460 breast cancer patients in the Republic of Korea (11), half were found to have sarcopenia, a somewhat higher prevalence than has been observed in the United States and France (10). Separately, a sarcopenia prevalence of 20.2% in Korean women 50 years and older in the general population was reported by a Korean National Health and Nutrition Examination Survey (12). This suggests that breast cancer patients may be more vulnerable to sarcopenia, and that the epidemiological status of sarcopenia and its prevalence in breast cancer patients require examination.

Determination of sarcopenia status involves assessment of alterations in body composition. Changes in body composition have been reported to occur in breast cancer patients during chemotherapy and endocrine therapy (9). In addition, obesity is known to be an accelerator of breast cancer, and the interaction between obesity and sarcopenia accelerates tumor recurrence (9). However, both clinical practice and oncology research assessments tend to focus on body mass index (BMI) or body surface area (BSA) rather than muscle mass change during treatment. Traditionally, BSA has been used to calculate chemotherapy doses, while BMI, a related measure that also incorporates weight and height, has often been employed in oncology research. In the last decade, the importance of identifying specific body composition changes during chemotherapy has emerged as an issue of interest (13–16). A 2018 study involving 119 breast cancer patients found sarcopenia status to be an independent prognostic factor for disease-free survival, while BMI was not significantly related to disease-free survival (17). The 2016 study of 1,460 Korean breast cancer patients showed that muscle volume was a significant prognostic factor for overall survival regardless of BMI, whereas fat volume and BMI were not significantly related to survival (11). Previous studies (11, 17) also have indicated that, unlike BMI, muscle mass appears to be a sensitive marker of overall survival. These studies show the importance of investigating body composition change during chemotherapy.

Although urgently needed, research in the area of body composition change during neoadjuvant chemotherapy for breast cancer is generally lacking. Descriptive analyses of body composition during other forms of cancer treatment (such as surgery, radiotherapy, or adjuvant chemotherapy) have been performed, for various cancer types (18–20), but few studies have examined changes in muscle mass during neoadjuvant chemotherapy among patients with breast cancer. This study was conducted to improve understanding of the current status of sarcopenia in breast cancer patients. Specifically, we evaluated body composition changes during neoadjuvant chemotherapy using CT imaging, assessed sarcopenia prevalence before and after neoadjuvant chemotherapy, and determined predictors of sarcopenia status in breast cancer patients after neoadjuvant chemotherapy.

## Materials and methods

### Study design and setting

We used a retrospective, descriptive, observational study design to determine body composition change and sarcopenia prevalence among breast cancer patients who had visited the breast cancer clinic at Severance Hospital in Seoul, Korea, from January 2017 to November 2020. Eligible participants were Korean women 20 years or older who had received a diagnosis of breast cancer at the hospital, completed neoadjuvant chemotherapy, and received at least two abdominal CT scans, before and after neoadjuvant chemotherapy, that provided images of the third lumbar spine vertebra (L3). We excluded patients with stage IV breast cancer and patients with CT images that were insufficient or inappropriate for analysis of body composition. Our sample size of 317 cases was sufficient to meet the study objectives. Initially, the target sample size was determined using G*Power 3.1 software. To accommodate regression analysis and pseudo R2 (0.39), a required sample size of 265 was calculated based on an odds ratio of 1.3. a power of 0.8.

Approval for this study was obtained from the Severance Hospital Institutional Review Board (#4-2021-0452) at Yonsei University Health Systems, Seoul, Korea. The Severance Hospital Data Review Board also approved the study data set with respect to protection of patients’ data and assessment of data-protection risks and tools.

### Data collection

The Big Data Team in the Yonsei University Health System provided comprehensive information from the selected sample’ medical records, including sociodemographic data and clinical data such as cancer- and treatment-related information. We used this information to assess age, clinical stage, TN stage, Ki-67, breast cancer subtype, chemotherapy regimen, duration of neoadjuvant chemotherapy, and BMI and BSA (both of which are calculated from weight and height). For Ki-67, we used a cutoff value based on the expressed cell ratio (<14% and ≥14%). For breast cancer subtype, we evaluated both hormone receptor and human epidermal growth factor receptor 2 (HER2); to explore HER2 gene amplification, we also assessed silver stain hybridization *in situ* findings.

**Figure 1:**
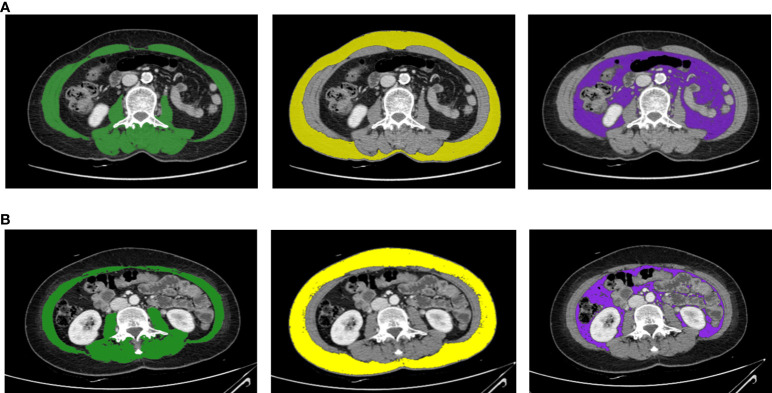
Body composition evaluation CT images for two breast cancer patients with the same BMI (23.37 kg/m^2^) and weight (54.7 kg). These axial CT images of the third lumbar vertebral region show that two study participants with the same BMI can have different body compositions. Panel **(A)** is a CT image for a 69-year-old female, and panel **(B)** is a CT image for a 48-year-old female. The images illustrate the different proportions of skeletal muscle mass (green), subcutaneous fat area (yellow), and visceral fat area (purple).

We measured body composition by analyzing CT images taken before and after chemotherapy. The Yonsei University Convergence Medical Technology Center performed the body composition analysis using Aquarius iNtuition viewer version 4.4.13.P6 (TeraRecon, Durham, North Carolina). Using the analysis program provided in the software, we analyzed the CT slices at L3 as a standardized landmark. We calculated the total cross-sectional areas of skeletal muscle, subcutaneous fat, and visceral fat (in cm^2^). The Hounsfield unit values used for measurement ranged from −29 to 150 for muscle, −190 to −30 for subcutaneous fat, and −150 to −50 for visceral fat. **Figure 1** presents a single axial CT slice at the L3 level and shows the areas of skeletal muscle, subcutaneous fat, and visceral fat for two study participants; this figure illustrates differences in body composition between two participants with the same BMI. The skeletal muscle index (SMI, in cm^2^/m^2^) was calculated as skeletal muscle area (in cm^2^) divided by height squared (in m^2^); both subcutaneous fat area and visceral fat area were also divided by height squared to determine the associated indexes. With respect to the sarcopenia cutoff value, we applied the value introduced by Prado et al. (SMI <38.5 cm^2^/m^2^) as an SMI cutoff for women (21).

Our investigative focus was on secondary sarcopenia, which involves loss of muscle mass—but not necessarily muscle function—accompanying cancer and other diseases (22). A variety of sarcopenia definitions and body composition evaluation techniques have been employed to assess sarcopenia across the international research. Investigation of primary sarcopenia, which is associated with aging, typically involves measurement of a combination of muscle mass, muscle strength, and physical performance. However, given that only muscle mass data were available for our retrospective study, we elected to concentrate our efforts on secondary sarcopenia, which can be evaluated through measurement of muscle mass alone.

### Statistical analysis

Data analysis was performed using Stata/IC 16 statistical software (College Station, Texas). We conducted descriptive analyses to characterize the study sample and to assess their body composition before (at baseline) and at completion of neoadjuvant chemotherapy. In addition, we used a paired *t*-test to compare sarcopenia prevalence and body composition change (i.e., changes in muscle mass and fat mass) before and after neoadjuvant chemotherapy. We calculated Pearson correlation coefficients to evaluate associations among body composition parameters. Finally, we applied multivariate logistic regression to determine predictors of sarcopenia at completion of neoadjuvant chemotherapy. All *p* values were two-tailed, and *p* values <.05 were considered significant.

## Results

### Demographic and clinical characteristics

**Table 1** presents descriptive data for the 317 women with breast cancer patients at baseline—before neoadjuvant chemotherapy. The mean age was 53 years, with ages ranging from 26 to 82 years. According to the BMI for Asia classification (23, 24), the most common weight category in the sample was normal (BMI of 18.5–22.9; *n* = 132, 41.6%), followed by obese (*n* = 94, 29.7%) and overweight (*n* = 82, 25.9%).

**Table 1:** Demographics and clinical characteristics of the sample (*N* = 317).

Characteristic	Category	Mean ± SD (range) or *n* (%)
Age (years)		52.78 ± 10.41 (26–82)
	20–29	3 (0.95)
	30–39	31 (9.78)
	40–49	93 (29.34)
	50–59	107 (33.75)
	≥60	83 (26.18)
Stage of tumor	I	6 (1.96)
	II	224 (73.20)
	III	76 (24.84)
Initial clinical T stage	1	30 (9.86)
	2	212 (69.74)
	3	32 (10.53)
	4	29 (9.54)
Initial clinical N stage	0	114 (37.50)
	1	148 (48.68)
	2	19 (6.25)
	3	23 (7.57)
Ki-67	Low (<14%)	34 (11.26)
	High (≥14%)	268 (88.75)
Tumor subtype	HR+/HER2−	108 (34.07)
	HR+/HER2+	49 (15.46)
	HR−/HER2+	58 (18.30)
	TNBC	102 (32.18)
Chemotherapy regimen	AC-T regimen	214 (67.51)
	TCHP regimen	84 (26.50)
	Other	19 (5.99)
Duration of neoadjuvant chemotherapy	(days)	142 ± 30.86 (49–196)
BMI at baseline	<18.5 (underweight)	9 (2.84)
	18.5–22.9 (normal)	132 (41.64)
	23–24.9 (overweight)	82 (25.87)
	≥25 (obese)	94 (29.65)

AC-T regimen, combination of an anthracycline and cyclophosphamide, followed by a taxane; BMI, body mass index; HER2−, human epidermal growth factor receptor 2-negative; HER2+, human epidermal growth factor receptor 2-positive; HR−, hormone receptor-negative; HR+, hormone receptor-positive; TCHP regimen, docetaxel, carboplatin, trastuzumab, and pertuzumab; TNBC, triple negative breast cancer.

Among the combinations of chemotherapy drugs used, the combination of anthracyclines and taxanes was the most common (*n* = 214, 67.5%), followed by the docetaxel, carboplatin, trastuzumab, and pertuzumab regimen (*n* = 84, 26.5%). The mean duration of chemotherapy was 142 days, with durations ranging from 49 to 196 days. Stage II cancer was the most common in the sample (*n* = 224, 73.2%).

### Body composition changes during neoadjuvant chemotherapy

**Figure 2:**
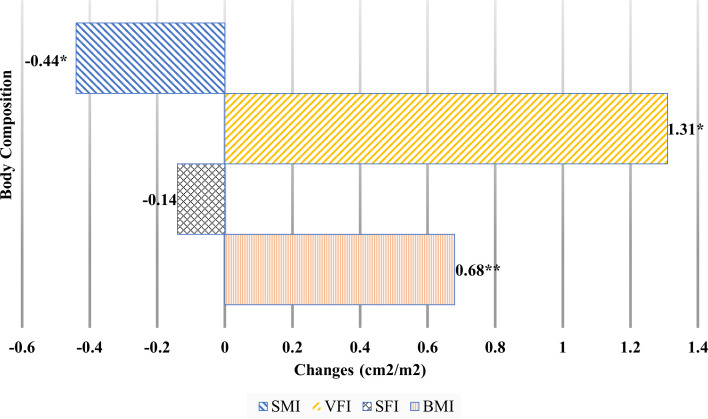
Body composition changes during neoadjuvant chemotherapy.BMI, body mass index; SFI, subcutaneous fat index; SMI, skeletal muscle mass index; VFI, visceral fat index. *p <.05, **p *<.01*.

**Table 2** and **Figure 2** show body composition change during neoadjuvant chemotherapy. Weight significantly increased (by an average of 0.67 kg) during neoadjuvant chemotherapy (from 59.33 to 60.00 kg, *t* (316) = −3.84, *p* = .001). Similarly, BMI and BSA (both based on weight in relation to their height) showed significant increases after neoadjuvant chemotherapy, by an average of 0.24 kg/m^2^ for BMI and 0.01 m^2^ for BSA.

**Table 2:** Body composition changes during neoadjuvant chemotherapy.

Parameter	Mean ± SD before NAC	Mean ± SD after NAC	*t*	*p* value
BMI	23.61 ± 3.17	23.85 ± 3.21	−3.315	0.001
Weight	59.33 ± 8.11	60.00 ± 8.53	−3.843	0.0001
BSA	1.61 ± 0.12	1.62 ± 0.13	−3.811	0.0002
SFI (cm^2^/m^2^)	66.58 ± 26.23	66.44 ± 26.57	0.190	0.849
VFI (cm^2^/m^2^)	29.97 ± 20.20	31.28 ± 19.15	−2.547	0.0114
SMI (cm^2^/m^2^)	42.37 ± 5.41	41.93 ± 5.76	2.153	0.0321

BMI, body mass index; BSA, body surface area; NAC, neoadjuvant chemotherapy; SFI, subcutaneous fat index; SMI, skeletal muscle mass index; VFI, visceral fat index.

Among the body composition changes during neoadjuvant chemotherapy, visceral fat index (VFI) significantly increased by 1.31 cm^2^/m^2^ (from 29.97 to 31.28 cm^2^/m^2^). There was no significant difference in subcutaneous fat index (SFI) before and after chemotherapy. SMI showed the only significantly decrease during neoadjuvant chemotherapy, by 0.44 cm^2^/m^2^ (from 42.37 to 41.93 cm^2^/m^2^).

### Sarcopenia prevalence during neoadjuvant chemotherapy

**Table 3** shows the sarcopenia prevalence before (at baseline) and at completion of neoadjuvant chemotherapy. According to Prado’s sarcopenia criteria (21), at baseline, 80 patients (25.24%) had sarcopenia and 237 patients (74.76%) did not. After neoadjuvant chemotherapy, the number of patients with sarcopenia increased (*n* = 93, 29.34%). Of the 80 patients who had sarcopenia before their chemotherapy, 64 (80%) retained their sarcopenia status after chemotherapy.

**Table 3:** Sarcopenia prevalence during neoadjuvant chemotherapy.

Group		After chemotherapy		
		No sarcopenia	Sarcopenia	Total
Before chemotherapy	No sarcopenia	208 (92.86%)	29 (31.18%)	237 (74.76%)
	Sarcopenia	16 (7.14%)	64 (68.62%)	80 (25.24%)
	Total	224 (100%)	93 (100%)	317 (100%)

Pearson χ^2^(1) = 132.48, p = 0.000.

### Associations between body composition changes

**Table 4** presents the statistically significant correlations between body composition parameters at baseline and the mean differences in these parameters after neoadjuvant chemotherapy. A lower SMI at baseline was associated with a greater loss of muscle mass during neoadjuvant chemotherapy (*r* = −.24, *p* <.001). Baseline SFI and VFI showed a similar direction of association with the mean differences in body composition parameters; lower VFI at baseline was associated with a greater loss of subcutaneous fat and visceral fat mass during neoadjuvant chemotherapy (*r* = −.22, *p* <.001 and *r* = −.34, *p* <.001, respectively). All mean differences in body composition parameters—including muscle, subcutaneous fat, and visceral fat mass—after neoadjuvant chemotherapy were positively related to one another. All significant relationships between body composition parameters were observed both at baseline and at completion of neoadjuvant chemotherapy.

**Table 4:** Associations between body composition changes.

Variables		Mean differences		
		SMI	SFI	VFI
Baseline	SMI	−.2381**	−.1048	−.1244*
	SFI	−.0363	−.2283**	−.0809
	VFI	−.0948	−.2202**	−.3387**

SFI, subcutaneous fat index; SMI, skeletal muscle mass index; VFI, visceral fat index. *p <.05, **p <.001.

### Multivariate logistic regression analyses to predict sarcopenia after neoadjuvant chemotherapy

As shown in **Table 5**, we fit a multivariate logistic regression model to predict sarcopenia status after neoadjuvant chemotherapy. The regression included patient age, tumor subtype, chemotherapy duration, chemotherapy regimen, baseline SFI and VFI, and baseline sarcopenic status as independent predictors (χ^2^[9] = 140.67, *p* = .0000, pseudo *R* ^2^ = 0.3908). Baseline sarcopenia status was the only significant predictor of sarcopenia status after neoadjuvant chemotherapy. Specifically, the sarcopenia after neoadjuvant chemotherapy increased for the group with baseline sarcopenia, with 3.357 times increasing log odds (β = 3.357,*p* <.001). Other variables showed no significant association with sarcopenia risk after neoadjuvant chemotherapy. Finally, no interactions of baseline sarcopenia status with other variables were observed to predict sarcopenia status after neoadjuvant chemotherapy.

**Table 5:** Multivariate logistic regression analyses to predict sarcopenia after neoadjuvant chemotherapy.

Variable	Value	Coef.	95% CI		*p-value*
Baseline sarcopenia	Yes	3.357	2.565	4.149	.000
	No	1.000			
Baseline subcutaneous fat		-.007	-.027	.013	.485
Baseline visceral fat		-.027	-.056	.001	.058
Age at diagnosis		.008	-.030	.046	.675
Chemotherapy regimen	AC-T regimen	1.000			
	TCHP regimen	2.089	-.237	4.415	.078
Tumor subtype	HR+/HER2−	1.000			
	HR+/HER2+	-.204	-1.847	1.439	.808
	HR−/HER2+	-.494	-2.171	1.183	.564
	TNBC	.091	-.781	.963	.838
Chemotherapy duration		.014	-.017	.045	.368

AC-T regimen, combination of an anthracycline and cyclophosphamide, followed by a taxane; CI, confidence interval; HER2−, human epidermal growth factor receptor 2-negative; HER2+, human epidermal growth factor receptor 2-positive; HR−, hormone receptor-negative; HR+, hormone receptor-positive; TCHP regimen, docetaxel, carboplatin, trastuzumab, and pertuzumab; TNBC, triple negative breast cancer. LR χ^2^(9) = 140.67, p <.000, pseudo R^2^ = 0.3908.

## Discussion

We conducted this study to improve understanding of the current status of sarcopenia in breast cancer patients by evaluating body composition changes during neoadjuvant chemotherapy, using CT imaging to assess sarcopenia prevalence before and after neoadjuvant chemotherapy, and determining predictors of sarcopenia status after neoadjuvant chemotherapy in this population. To our knowledge, this is the first study to use CT imaging to comprehensively quantify body composition changes during neoadjuvant chemotherapy in Korean breast cancer patients. Determination of sarcopenia status is often complicated or obscured by obesity; sarcopenia is difficult to recognize visually without medical imaging. Therefore, this study used abdominal CT scans, which are routinely performed during treatment and are among the most accurate measures of body composition parameters, to accurately quantify body composition changes.

Our study generated meaningful findings regarding sarcopenia status and body composition changes among patients with breast cancer undergoing neoadjuvant chemotherapy. Notably, BMI and BSA increased significantly during neoadjuvant chemotherapy. This finding is clinically significant because BSA is typically used in clinical oncology settings to calculate chemotherapy doses. Additionally, an unchanged BSA during chemotherapy can mask more specific changes to body composition, such as alterations in the proportions of fat and muscle. For example, the detailed body composition results in our study showed that although visceral fat mass significantly increased during neoadjuvant chemotherapy, SMI significantly decreased. While the observed weight gain indicates that most body composition–related variables increased or were maintained, that gain can also mask significant decreases in the actual amount of skeletal muscle mass. These findings suggest that weight maintenance and muscle mass maintenance during chemotherapy are not proportional, and thus that muscle mass loss could be easily overlooked in clinical settings.

Among the important findings of this study, 80% of the patients who had sarcopenia before neoadjuvant chemotherapy (*n* = 64) maintained their sarcopenia status after completing chemotherapy. Additionally, 12.2% of those who did not have sarcopenia at baseline (*n* = 29) were newly diagnosed with the condition after neoadjuvant chemotherapy. A similar result was reported by a systematic review and meta-analysis involving 11 studies of patients receiving neoadjuvant therapy for esophageal cancer, which found that 15.4% of participants showed new incidence of sarcopenia after neoadjuvant chemotherapy (25). Another multi-institutional analysis, involving patients with gastric cancer, showed that 14% had newly developed sarcopenia during neoadjuvant chemotherapy (26). In comparison, a previous systematic review and meta-analysis in a general population found that 10% of women aged 60 years or older had sarcopenia and that non-Asian women had a higher prevalence of sarcopenia than Asian women (20% versus 11%), as measured by bioelectric impedance analysis (27).

Considering that the overall sarcopenia prevalence in Korean women 50 years and older has been reported as about 20% (12), it is noteworthy that even before surgery, almost 30% of the breast cancer patients in our study (mean age = 53 years) were diagnosed with sarcopenia. Our findings, combined with those of previous studies, show that the prevalence of newly diagnosed sarcopenia in breast cancer patients after neoadjuvant chemotherapy is similar to that in patients with other types of cancer. However, the overall prevalence of sarcopenia in breast cancer patients is higher than that in the healthy population. Therefore, careful assessment of breast cancer patients for sarcopenia—with consideration of cancer type, cultural background, age, and treatment- and cancer-related factors—is necessary.

Among our findings, baseline sarcopenia status was the only significant predictor of sarcopenia after neoadjuvant chemotherapy. The dramatic rise in the risk of sarcopenia after neoadjuvant chemotherapy in the baseline sarcopenia group— with 3.357 times increasing log odds —is a remarkable finding that has important implications for assessment of muscle mass during treatment. Many studies have shown that preoperative sarcopenia is a risk factor for postoperative complications, severe complications, decreased overall survival, and decreased disease-free survival in patients with various cancer types (28–33). Given that preoperative sarcopenia status appears to be a significant prognostic factor for poor treatment outcomes and reduced survival, our findings indicate that ongoing assessment of sarcopenia as well as overall body composition from the beginning of cancer treatment could enhance long-term therapeutic effectiveness for breast cancer patients.

Several recent studies on sarcopenia, or muscle mass loss, have drawn the attention of the scientific community because they identified muscle mass loss as a key factor in chemotherapy toxicity and hospitalization as well as mortality for various cancer types (34–38). Previous studies have reported that BMI is not related to breast cancer progression but that muscle volume is a significant factor influencing severe laboratory adverse events (8) and overall survival (11). In clinical settings, therefore, combined assessments of sarcopenia and body composition change would be beneficial for predicting patients’ chemotherapy toxicities and mortality. Such assessments would also support early intervention to prevent or reduce muscle mass loss. Thus, to fully understand breast cancer patients’ condition in the oncology setting, combined interpretations of various body composition changes are called for; such interpretations can reveal patients’ sarcopenia status, provide direction for personalized chemotherapy, and help to prevent chemotherapy toxicity.

### Study limitations

This retrospective study had limitations that should be acknowledged. First, we focused on changes in body composition during neoadjuvant chemotherapy, which prevented us from examining the trajectory of sarcopenia during subsequent surgery and radiotherapy. Future studies should apply a repeated measures design throughout the entire process of active cancer treatment to obtain a better understanding of patients’ sarcopenia status and trajectory. Second, because the data available to us were limited by our retrospective design, we could not examine potential mediators for sarcopenia (such as nutrition and exercise) during neoadjuvant chemotherapy. Furthermore, the retrospective data available to us were limited to muscle mass measurements, as muscle strength and physical performance data had not been collected. Consequently, we focused our evaluation on secondary sarcopenia, for which muscle mass data would suffice. Based on the available evidence on various factors related to sarcopenia, future studies should comprehensively investigate potential mediators, their associations with sarcopenia, and their underlying mechanisms, with the goal of minimizing muscle mass loss during cancer treatment. Finally, as sarcopenia is more prevalent in the general population of Korea than is the case in the United States and Europe, our study sample may have been subject to selection bias.

### Conclusion

Comprehensive body composition analysis can improve our understanding of subtle yet meaningful changes in muscle and fat mass among breast cancer patients undergoing neoadjuvant chemotherapy. To reduce masking of muscle mass loss during cancer treatment in clinical settings, active interpretation of body composition beyond BSA and BMI assessment is clearly needed. This is particularly true for patients who have sarcopenia when they begin neoadjuvant chemotherapy, as their risk of muscle mass loss during treatment is alarmingly high. Assessment of body composition and sarcopenia status beginning at breast cancer diagnosis and extending throughout the neoadjuvant chemotherapy period should become a cornerstone for successful completion of planned cancer treatment.

## Data availability statement

The raw data supporting the conclusions of this article will be made available by the authors, without undue reservation.

## Ethics statement

This study was reviewed and approved by the Severance Hospital Institutional Review Board in the Yonsei University Health Systems, Seoul, Korea (#4-2021-0452). Written informed consent for participation was not required for this study in accordance with the national legislation and the institutional requirements. Written informed consent was not obtained from the individual(s) for the publication of any potentially identifiable images or data included in this article.

## Author contributions

MJ, SK, SP, and JG, study concept and design. MJ and SK, data acquisition and data quality control. MJ and CP, data analysis and interpretation, manuscript preparation. MJ, SK, SP, CP, JG, and AD, contributed to the article, reviewed the manuscript, and approved the submitted version. All authors contributed to the article and approved the submitted version.

## Funding

This work was supported by a National Research Foundation of Korea (NRF) grant, which was funded by the government of the Republic of Korea through the Ministry of Science and ICT (NRF No. 2021R1C1C2004628).

## Conflict of interest

The authors declare that the research was conducted in the absence of any commercial or financial relationships that could be construed as a potential conflict of interest.

## Publisher’s note

All claims expressed in this article are solely those of the authors and do not necessarily represent those of their affiliated organizations, or those of the publisher, the editors and the reviewers. Any product that may be evaluated in this article, or claim that may be made by its manufacturer, is not guaranteed or endorsed by the publisher.

## Abbreviations

BMI, body mass index; BSA, body surface area; L3, third lumbar spine vertebra; SFI, subcutaneous fat index; SMI, skeletal muscle index; VFI, visceral fat index.
